# Body mass index and waist-to-height ratio effect on mortality in non-alcoholic fatty liver: revisiting the obesity paradox

**DOI:** 10.3389/fendo.2024.1419715

**Published:** 2024-12-06

**Authors:** Hao Jiang, Mingkai Li, Hongsheng Yu, Yinan Huang, Bilan Yang, Bin Wu, Yidong Yang

**Affiliations:** ^1^ Department of Gastroenterology, the Third Affiliated Hospital of Sun Yat-Sen University, Guangzhou, China; ^2^ Guangdong Provincial Key Laboratory of Liver Disease Research, Guangzhou, Guangdong, China; ^3^ Gastrointestinal Endoscopy Center, the Eighth Affiliated Hospital of Sun Yat-sen University, Shenzhen, China

**Keywords:** anthropometrics, body mass index, mortality, overweight, waist-to-height ratio

## Abstract

**Purpose:**

Emerging research indicates that individuals with non-alcoholic fatty liver disease (NAFLD) who carry excess weight have similar or even higher survival rates than their normal-weight counterparts. This puzzling “obesity paradox” may be attributed to underlying biases. To explore this phenomenon, we examined data extracted from the third National Health and Nutrition Examination Survey (NHANES) III, which spanned from 1988-1994.

**Methods:**

We specifically targeted participants diagnosed with NAFLD through ultrasound due to fatty liver presence and employed multivariate Cox regression to assess mortality risk associated with body mass index (BMI) and the waist-to-height ratio (WHtR).

**Results:**

Over a median follow-up period of 20.3 [19.9-20.7] years, 1832 participants passed away. The study revealed an intriguing “obesity−survival paradox”, in which individuals classified as overweight (HR 0.926, 95% CI 0.925–0.927) or obese (HR 0.982, 95% CI 0.981–0.984) presented reduced mortality risks compared with those categorized as normal weight. However, this paradox vanished upon adjustments for smoking and exclusion of the initial 5-year follow-up period (HR 1.046, 95% CI 1.044–1.047 for overweight; HR 1.122, 95% CI 1.120–1.124 for obesity class I). Notably, the paradox was less pronounced with the WHtR, which was significantly different only in quartile 2 (HR 0.907, 95% CI 0.906–0.909) than in quartile 1, and was resolved after appropriate adjustments. In particular, when BMI and WHtR were considered together, higher levels of adiposity indicated a greater risk of mortality with WHtR, whereas BMI did not demonstrate the same trend (*p <*0.05).

**Conclusion:**

The “obesity paradox” in NAFLD patients can be explained by smoking and reverse causation. WHtR was a better predictor of mortality than BMI.

## 1 Introduction

The global incidence of overweight and obesity has increased and continues to rise (1, 2). The World Health Organization (WHO) has estimated the number of overweight adults globally at over 2.5 billion, categorizing overweight as a body mass index (BMI) between 25–30 kg/m², and identified more than 890 million individuals as obese, with a BMI of 30 kg/m² or higher. Between 2000 and 2018, the prevalence of obesity in the United States increased from 30.5% to 42.4%. Even more alarming is the fact that, within the same timeframe, the incidence of extreme obesity almost doubled, jumping from 4.7% to 9.2% (3).

Comprehensive evaluations of extensive, long-term prospective studies typically reveal a correlation between increased mortality rates and both obesity and overweight conditions, as well as being underweight, which the WHO defines as a BMI under 18.5 kg/m² (4, 5). In the past few years, the term “obesity paradox” has become increasingly common in the literature (6, 7), referring to the observed phenomenon whereby individuals who are obese or overweight seem to have increased survival rates. The phrase “obesity paradox” first emerged in the medical literature in 2002, within the title of a paper by Gruberg and colleagues. Their research concluded that normal-weight patients presented the greatest likelihood of complications during hospital stays, cardiac fatalities, and increased mortality following procedures such as percutaneous coronary interventions (8). A PubMed search for the “obesity paradox” dated November 9, 2023, revealed a total of 2,199 publications, indicating a significant uptrend over time.

NAFLD is a widespread chronic liver condition that affects an estimated 17–46% of people worldwide, and it also occurs in approximately 7% of individuals who are normal weight (9). Recent guidelines from the American Gastroenterological Association, along with numerous publications, have highlighted that “lean individuals” [BMI <25 kg/m² (non-Asian populations) or BMI <23 kg/m² (Asian populations)] with NAFLD exhibit comparable or even greater cardiac metabolic risk factors, risk assessments, incidences of cardiovascular events, and mortality rates than their counterparts who are overweight or obese with NAFLD (10, 11). This phenomenon is often referred to as the “obesity paradox.”

However, some experts argue that the “obesity paradox” is a misnomer, suggesting that it dissipates when factors such as smoking and reverse causation are accounted for (12, 13). Reverse causation implies situations where an individual’s weight is the result of an illness rather than the cause. Disease often leads to reduced weight due to a diminished appetite or increased energy expenditure, which is correlated with increased mortality rates. Given that weight loss is more prevalent among sick individuals and that mortality rates are elevated, reverse causation poses a significant challenge for accurately assessing the mortality risks associated with obesity, especially when analyses focus solely on such populations (14). After controlling for smoking status and reversing causality, will the “obesity paradox” still exist in NAFLD patients?

BMI is widely recognized as the primary anthropometric measurement, yet it presents multiple shortcomings. It fails to consider factors such as body fat distribution and composition; skeletal mass; and the impacts of age, sex, and ethnicity. Consequently, alternative anthropometric indicators, including waist circumference, waist-to-hip ratio, and waist-to-height ratio, have been suggested. These measures more accurately represent the presence and quantity of ectopic fat, particularly in the abdominal area, which is known to be more metabolically active and detrimental than subcutaneous fat (15).

Recent research indicates that these alternative measures surpass BMI in forecasting the likelihood of chronic illnesses, various causes of death, cardiovascular diseases, and cancer mortality in the broader population. Despite their effectiveness, they have yet to be widely adopted in clinical settings (16).

Our research was designed to evaluate the relationships among BMI, WHtR, and the risk of mortality among patients with NAFLD while considering potential confounding factors and reverse causation. To reduce the impact of reverse causation on the association between obesity and mortality, we methodically adjusted for smoking habits; excluded subjects with less than five years of follow-up; and considered prevalent chronic conditions, mainly cardiovascular diseases, cancers, or chronic respiratory diseases.

## 2 Patients and methods

### 2.1 Study design

This research employs data from the ongoing National Health and Nutrition Examination Surveys (NHANES), which are managed by the U.S. National Center for Health Statistics. This was a retrospective analysis of a population-based cohort of NAFLD individuals using data from NHANES III; the survey was executed from 1988 to 1994. NHANES III encompasses a detailed dataset that utilizes a stratified, clustered, and multistage probability sampling approach to secure a representative segment of the noninstitutionalized civilian population in the U.S.

The questionnaire responses, including medical history and demographic details, were provided by the participants themselves. The NHANES aims to assess health and nutritional status within the U.S. by compiling demographic, dietary, physical examination, laboratory, and questionnaire information from both adults and children. Hepatic ultrasound examinations were also included in the NHANES III. Thus, NHANES III was selected for its application of ultrasound in defining fatty liver conditions. Additionally, the period during which NHANES III was conducted, from 1988 to 1994, allowed for the analysis of long-term mortality outcomes for the cohort. The Centers for Disease Control and Prevention’s researchers have endorsed the NHANES, with all participants providing informed consent.

### 2.2 Participants

**Figure 1:**
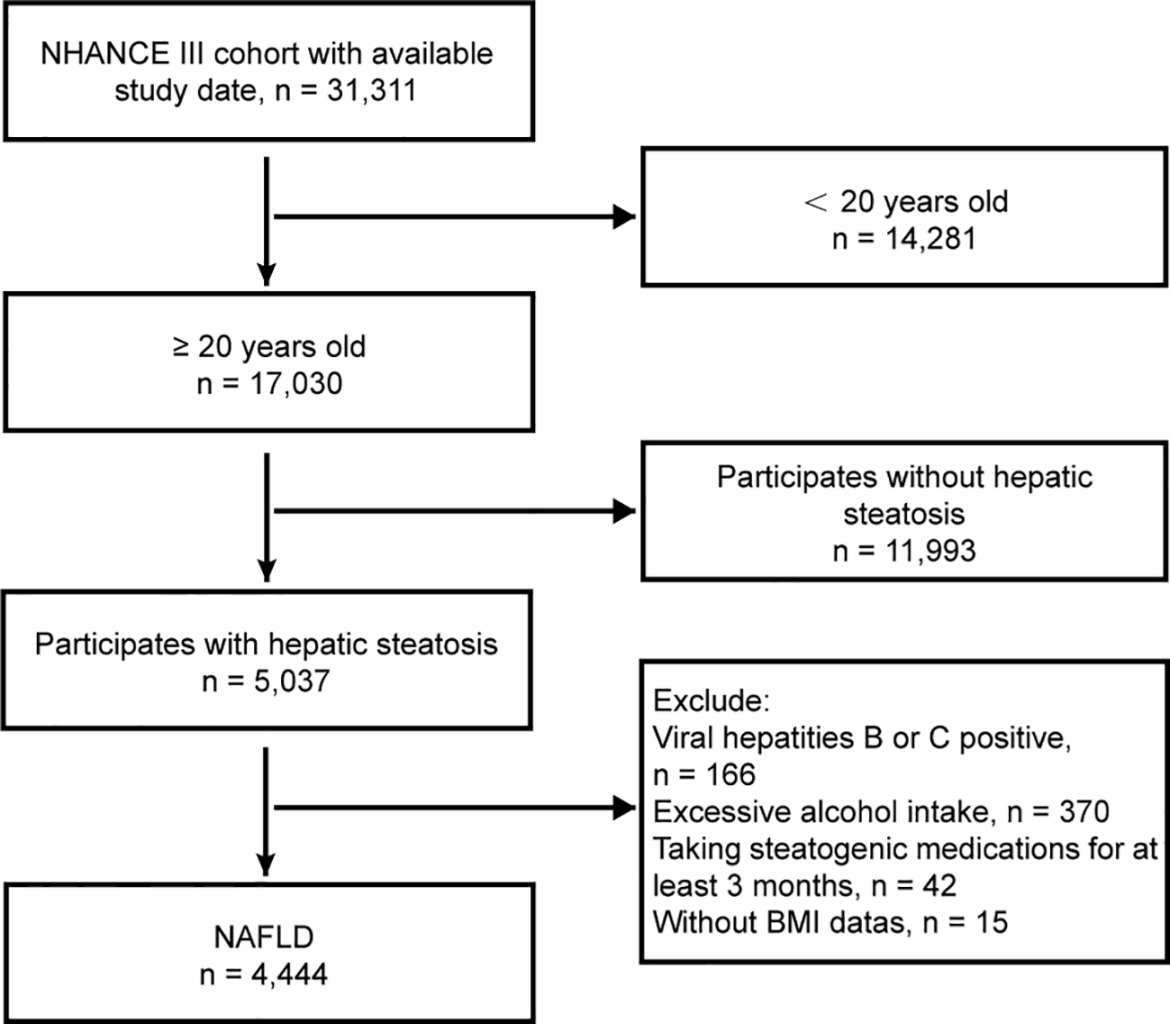
Flowchart of the study design.

The study reviewed demographic data, physical exams, laboratory tests, and questionnaire responses from 31,311 patients. Individuals aged 20 years and above who had comprehensive laboratory data and physical metrics necessary for a precise NAFLD diagnosis were included. The exclusion criteria were as follows: (1) absence of hepatic steatosis, (2) positive serology for hepatitis B or C, (3) high alcohol intake (>30 g/day for males, >20 g/day for females), (4) prolonged use of steatogenic medication (over 3 months) (see **Supplementary Table S1**), and (5) missing BMI information. The patient flowchart is shown in **Figure 1**.

### 2.3 Definition of hepatic steatosis

Hepatic steatosis identification in NHANES III subjects was performed via hepatic steatosis ultrasound examination. Adult participants underwent hepatic ultrasound at a mobile center via a Toshiba Sonolayer SSA-90A machine (Toshiba America Medical Systems, Inc., Tustin, California). Board-certified radiologists evaluated hepatic steatosis via five criteria: parenchymal brightness, contrast between the liver and kidney, deep beam attenuation, visibility of vessel walls, and clarity of the gallbladder wall. The ultrasound findings were classified as normal, mild, moderate, or severe hepatic steatosis. In accordance with quality control standards, reliability metrics (intra- and inter-rater) were established. The intra-rater reliability was 91.3% (kappa 0.77), and the inter-rater reliability was 88.7% (kappa 0.70).

#### 2.3.1 Other definitions and measurements

In this study’s BMI analysis, participants were classified following the World Health Organization (WHO) BMI categories as follows: underweight (<18.5 kg/m^2^), normal weight (18.5−24.9 kg/m^2^), overweight (25.0−29.9 kg/m^2^), and obesity classes I (30.0−34.9 kg/m2), II (35.0−39.9 kg/m2), and III (≥40 kg/m2) (16). The WHtR was divided into quartiles: <0.53, 0.53−0.60, 0.60−0.66, and >0.66, with a WHtR >0.6 signifying abdominal obesity. Diabetes is identified by fasting plasma glucose levels of ≥126 mg/dl or an HbA1c of ≥6.5% (17). Hypertension is defined as having a systolic blood pressure >130 mmHg, a diastolic pressure >85 mmHg, or being on antihypertensive drugs (18). Information on smoking habits and alcohol intake was gathered via self-administered questionnaires. Demographic and socioeconomic data such as age, gender, race, ethnicity (including Non-Hispanic White, Non-Hispanic Black, Mexican American, and Other Asian, which the NHANES did not oversample until 2012), education (no more than high school, beyond high school), and the poverty income ratio were collected. The participants also self-reported comorbidities such as stroke, heart attack, congestive heart failure, respiratory diseases, and cancer. Excessive alcohol consumption was defined as more than 30 g/day for men and 20 g/day for women.

Physical and blood parameters, including height, weight, waist circumference, BMI, WHtR, aspartate aminotransferase (AST), alanine aminotransferase (ALT), alkaline phosphatase (ALP), albumin, gamma-glutamyl transferase (GGT), globulin, glycosylated hemoglobin (HBA1C), high-density lipoprotein cholesterol (HDL-C), platelet count, and plasma fasting glucose and triglyceride levels, were measured by skilled medical staff following standardized procedures.

#### 2.3.2 Mortality follow-up

Vital status data from the NHANES were sourced from the national death index and are accessible in public use files by the National Center for Health Statistics, with comprehensive records up to December 31, 2018.

### 2.4 Statistical analyses

Weighted analyses utilized the NHANES survey weights to reflect the survey’s design, nonresponse, post-stratification, and oversampling. These weights ensure that our statistical estimates represent the U.S. civilian, noninstitutionalized populace.

Descriptive statistics are presented as weighted percentages for categorical variables and means with standard deviations for continuous variables. Chi-square tests were used to assess the distributions of categorical variables, such as sex, race, ethnicity, education, and comorbidities, among patients. Student’s t tests were performed to evaluate the distributions of continuous variables, including age, blood biochemical laboratory results, and physical measurements.

Multivariate Cox proportional hazards models were used to calculate hazard ratios (HRs) and 95% confidence intervals (CIs) for mortality across BMI and WHtR categories, referencing the normal weight group and the lowest WHtR quartile. Adjustments were made for age, sex, ethnicity, smoking, and baseline chronic diseases (hypertension, diabetes, heart failure, heart attack, stroke, asthma, chronic bronchitis, skin cancer, other cancers), and the first 5 years of follow-up were excluded to reduce confounding factors and minimize reverse causation risk, where a pre-existing condition influences both exposure and outcome. For example, NAFLD patients with severe liver disease or other comorbidities may experience weight loss or a reduced WHtR, potentially increasing their mortality risk. Additionally, mortality risks were determined for four groups based on BMI and WHtR: Non-obesity (BMI <30 kg/m², WHtR <0.6), Simple abdominal obesity (BMI <30 kg/m², WHtR ≥0.6), Simple general obesity (BMI ≥30 kg/m², WHtR <0.6), and Double obesity (BMI ≥30 kg/m², WHtR ≥0.6). Survival curves were generated via the Kaplan–Meier method and compared via the log-rank test. Data analysis was conducted via R (version 4.2.0) and EmpowerStats (version 4.1). All p values were two-tailed, with p < 0.05 considered statistically significant.

## 3 Results

### 3.1 Study population

A total of 31,311 participants from the NHANES III cohort (1988-1994) were initially included in the study. However, after 26,867 individuals were excluded, the final analysis was conducted on a sample size of 4,444 participants (**Figure 1**). The median age of the analyzed population was 45.25 years, with a range of 30.32 to 60.18 years. Among the participants, 49.62% were male, and 72.31% were classified as overweight or obese. The study recorded a total of 1,831 deaths, with 949 occurring in males and 882 in females.

### 3.2 Baseline characteristics of the participants

#### 3.2.1 Body mass index

In this analysis, the distribution of BMI among 71 patients was below 18.5 kg/m²; 1021 patients fell within the 18.5-24.9 kg/m² range; 1486 were categorized between 25-29.9 kg/m²; 1066 between 30-34.9 kg/m²; and 800 had a BMI of 35 kg/m² or greater. **Supplementary Table S2** presents the detailed baseline characteristics segmented by these BMI groups.

#### 3.2.2 Waist-to-height ratio

Patients were grouped into four quartiles based on their WHtR: 0.35 to 0.53, 0.53 to 0.60, 0.60 to 0.66, and 0.66 to 1.00 for the first through fourth quartiles, respectively. **Supplementary Table S3** outlines the baseline characteristics of patients according to WHtR quartiles.

### 3.3 Overlap between body mass index categories and waist-to-height ratio quartiles

**Figure 2:**
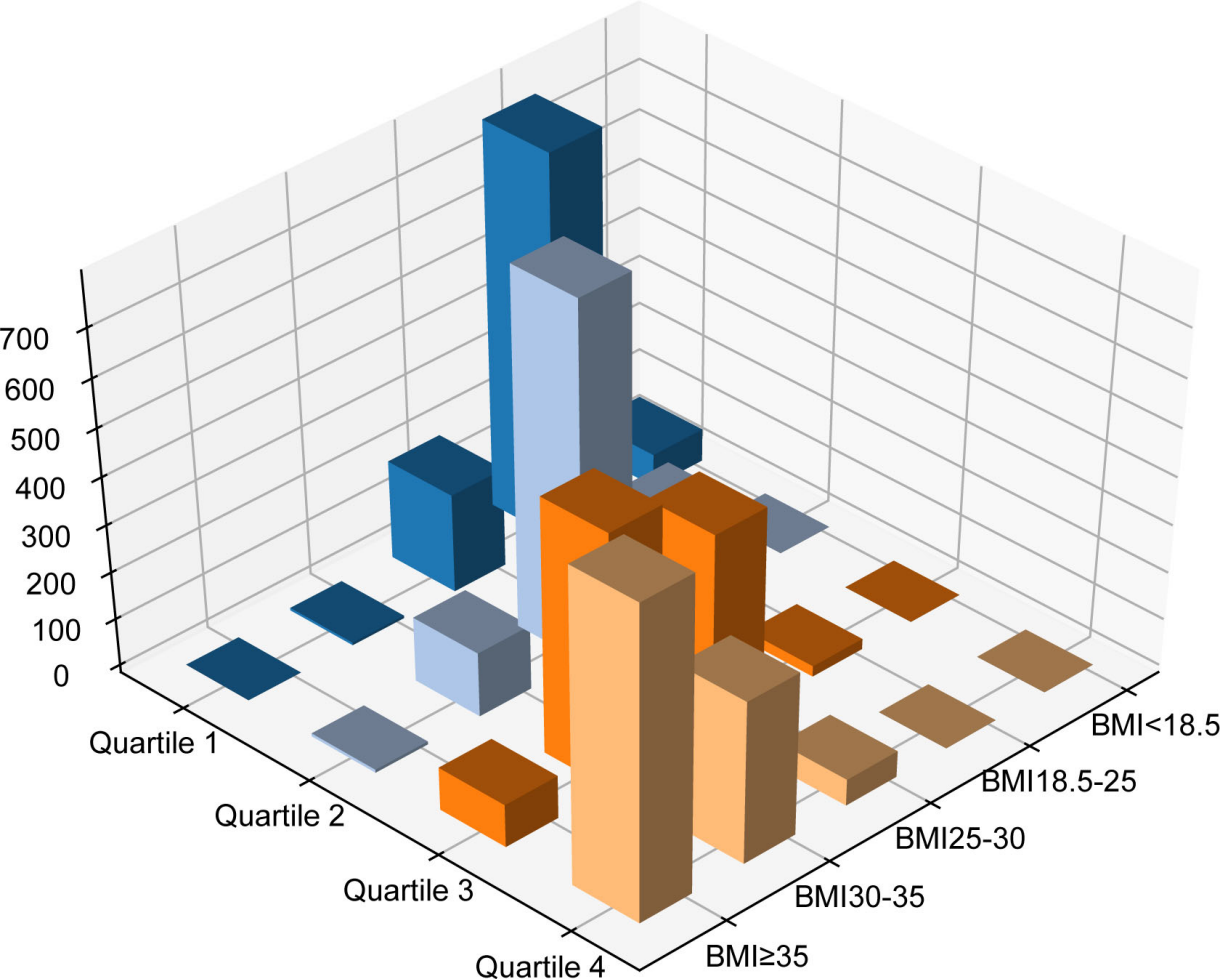
Different combinations of BMI and waist-to-height ratio. BMI, body mass index; Q, quartile of waist-to-height ratio.

**Table 1** and **Figure 2** illustrate the intersection between BMI categories and WHtR quartiles. Among the 1433 patients classified as overweight by BMI, 750 (52.3%) fell into the second WHtR quartile. The other 683 participants (47.7%) were in the first, third or fourth WHtR quartiles. Among the 1065 patients not classified as overweight or obese by BMI, 862 (80.94%) were in the first WHtR quartile, which is within the range considered healthy. Conversely, of the 1798 patients in obesity classes I or II/III, 1650 (91.77%) were in the third or fourth WHtR quartiles.

**Table 1:** Correlations between body mass index categories and waist-to-height ratios.

	Underweight BMI < 18.5	Normal weight BMI 18.5 - 25	Overweight BMI 25 - 30	Obesity class I BMI 30 - 35	Obesity class II/III BMI ≥ 35
WHtR Quartile 1	68 (100.0%)	794 (79.6%)	207 (14.4%)	5 (0.5%)	0 (0.0%)
WHtR Quartile 2	0 (0.0%)	181 (18.2%)	750 (52.3%)	137 (13.3%)	6 (0.8%)
WHtR Quartile 3	0 (0.0%)	22 (2.2%)	420 (29.3%)	540 (52.4%)	92 (12.0%)
WHtR Quartile 4	0 (0.0%)	0 (0.0%)	56 (3.9%)	348 (33.8%)	670 (87.2%)

Spearman correlation coefficient: 0.842.

### 3.4 Outcomes according to anthropometric measures

#### 3.4.1 Body mass index

**Figure 3:**
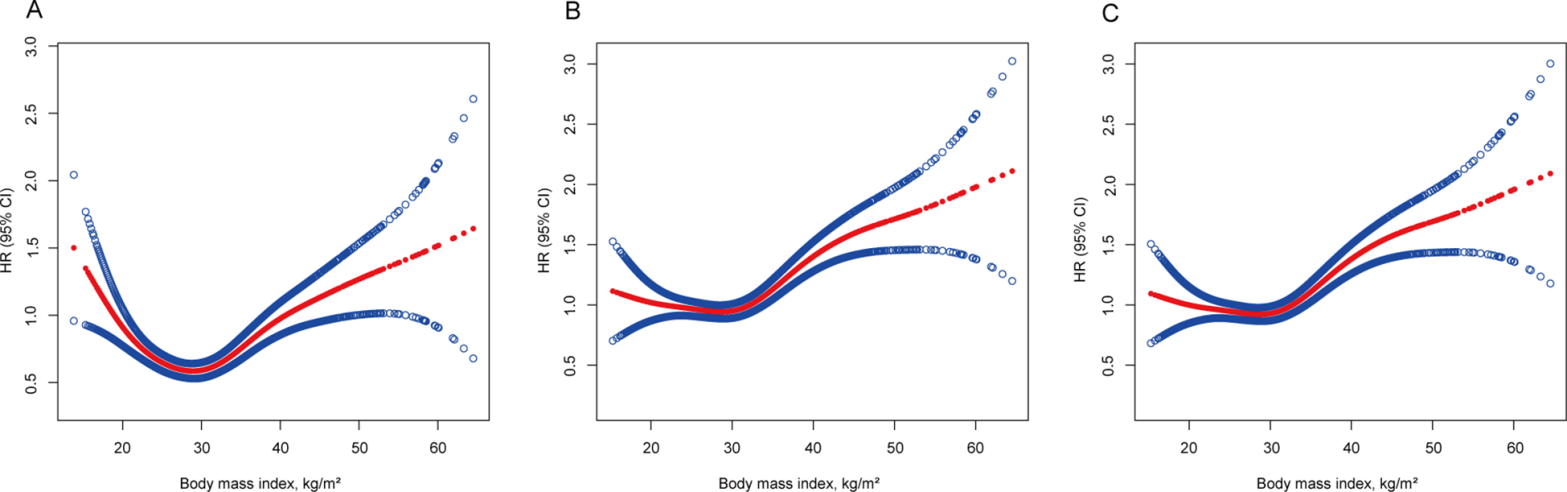
Association of body mass index with all-cause mortality. **(A)** Adjusted for age, sex and ethnicity. **(B)** Adjusted for age, sex, ethnicity, and smoking status and excluding the first 5 years of follow-up. **(C)** Adjusted for age, sex, ethnicity, smoking status, known chronic disease at baseline (hypertension, diabetes, heart failure, heart attack, stroke, asthma, chronic bronchitis, skin cancer, other cancers) and excluding the first 5 years of follow-up. The solid line represents the hazard ratio, and the dotted line represents the 95% CI. CI, confidence interval; HR, hazard ratio.

During the median follow-up period of 20.3 years (range 19.9–20.7 years), 1832 participants passed away. In the BMI analysis according to WHO category, the mortality risk was notably lower for overweight individuals (HR 0.926, 95% CI 0.925–0.927) and for obesity class I individuals (HR 0.982, 95% CI 0.981–0.984) when adjusted solely for age, sex, and ethnicity. However, this correlation disappeared after adjustments for smoking and the exclusion of the initial 5 years of follow-up (HR 1.046, 95% CI 1.044–1.047 for overweight; HR 1.122, 95% CI 1.120–1.124 for obesity class I), indicating that smoking and reverse causation significantly confounded the “obesity−survival paradox.” Further adjustments for known chronic diseases at baseline (hypertension, diabetes, heart failure, heart attack, stroke, asthma, chronic bronchitis, skin cancer, other cancers) did not substantially alter the outcomes (HR 1.018, 95% CI 1.017–1.020 for overweight; HR 1.094, 95% CI 1.092–1.095 for obesity class I), as shown in **Table 2**. A similar trend was observed when BMI was treated as a continuous variable (**Figure 3**).

**Table 2:** Outcomes according to body mass index.

	Underweight BMI < 18.5	Normal weight BMI 18.5 - 25	Overweight BMI 25 - 30	Obesity class I BMI 30 - 35	Obesity class II/III BMI ≥ 35
All-cause mortality					
Deaths/Participants (%)	21/71 (29.58%)	323/1021 (31.64%)	649/1486 (43.67%)	476/1066 (44.65%)	363/800 (45.36)
Event rate per 1000 person-years	12.87	13.29	19.48	19.71	20.15
HR (95% CI)^a^	2.673 (2.662, 2.683)	Reference	0.926 (0.925, 0.927)	0.982 (0.981, 0.984)	1.611 (1.609, 1.614)
HR (95% CI)^b^	2.112 (2.102, 2.122)	Reference	1.046 (1.044, 1.047)	1.122 (1.120, 1.124)	1.945 (1.941, 1.948)
HR (95% CI)^c^	1.968 (1.958, 1.977)	Reference	1.018 (1.017, 1.020)	1.094 (1.092, 1.095)	1.857 (1.854, 1.861)

BMI, body mass index; CI, confidence interval; HR, hazard ratio.

a Adjusted for age, sex and ethnicity.

b Adjusted for age, sex, ethnicity, and smoking status and excluding the first 5 years of follow-up.

c Adjusted for age, sex, ethnicity, smoking status, known chronic disease at baseline (hypertension, diabetes, heart failure, heart attack, stroke, asthma, chronic bronchitis, skin cancer, other cancers) and excluding the first 5 years of follow-up.

#### 3.4.2 Waist-to-height ratio

In the quartile-based analysis of the WHtR, quartile 2 had a significantly lower mortality risk (HR 0.907, 95% CI 0.906–0.909) than quartile 1 when adjusted only for age, sex, and ethnicity (**Table 3**). However, this association was negated (HR 1.058, 95% CI 1.056–1.060) after additional adjustments for smoking status and the exclusion of the initial 5-year follow-up. Further adjustments for baseline chronic diseases (hypertension, diabetes, heart failure, heart attack, stroke, asthma, chronic bronchitis, skin cancer, other cancers) still revealed a greater risk in Quartile 2 (HR 1.062, 95% CI 1.060–1.064) than in Quartile 1 (**Table 3**).

**Table 3:** Outcomes according to quartile of waist-to-height ratio.

	Quartile 1	Quartile 2	Quartile 3	Quartile 4
All-cause mortality				
Deaths/Participants (%)	238/1074 (22.16%)	418/1074 (38.92%)	529/1074 (49.26%)	563/1074 (52.42%)
Event rate per 1000 person-years	8.85	16.71	22.36	24.43
HR (95% CI)^a^	Reference	0.907 (0.906, 0.909)	1.189 (1.187, 1.191)	1.656 (1.654, 1.659)
HR (95% CI)^b^	Reference	1.058 (1.056, 1.060)	1.404 (1.402, 1.407)	1.948 (1.945, 1.952)
HR (95% CI)^c^	Reference	1.062 (1.060, 1.064)	1.381 (1.378, 1.383)	1.880 (1.877, 1.883)

CI, confidence interval; HR, hazard ratio.

a Adjusted for age, sex and ethnicity.

b Adjusted for age, sex, ethnicity, smoking status and excluding the first 5 years of follow-up.

c Adjusted for age, sex, ethnicity, smoking status, known chronic disease at baseline (hypertension, diabetes, heart failure, heart attack, stroke, asthma, chronic bronchitis, skin cancer, other cancers) and excluding the first 5 years of follow-up.

**Figure 4:**
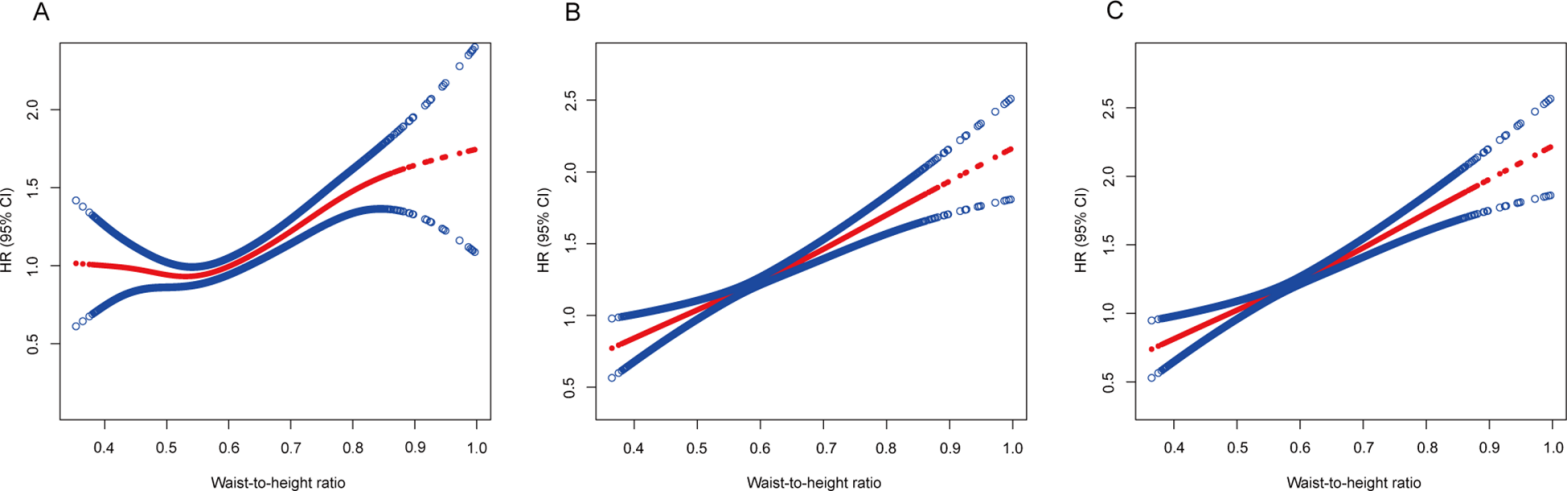
Association of the waist-to-height ratio with all-cause mortality. **(A)** Adjusted for age, sex and ethnicity. **(B)** Adjusted for age, sex, ethnicity, and smoking status and excluding the first 5 years of follow-up. **(C)** Adjusted for age, sex, ethnicity, smoking status, known chronic disease at baseline (hypertension, diabetes, heart failure, heart attack, stroke, asthma, chronic bronchitis, skin cancer, other cancers) and excluding the first 5 years of follow-up. The solid line represents the hazard ratio, and the dotted line represents the 95% CI. CI, confidence interval; HR, hazard ratio.

When the WHtR was assessed as a continuous measure, the trend was consistent. Post-adjustment for smoking and initial 5-year exclusion, the relationship transformed into an upward linear trend (**Figure 4**).

### 3.5 The combined effect of body mass index and the waist-to-height ratio

**Figure 5:**
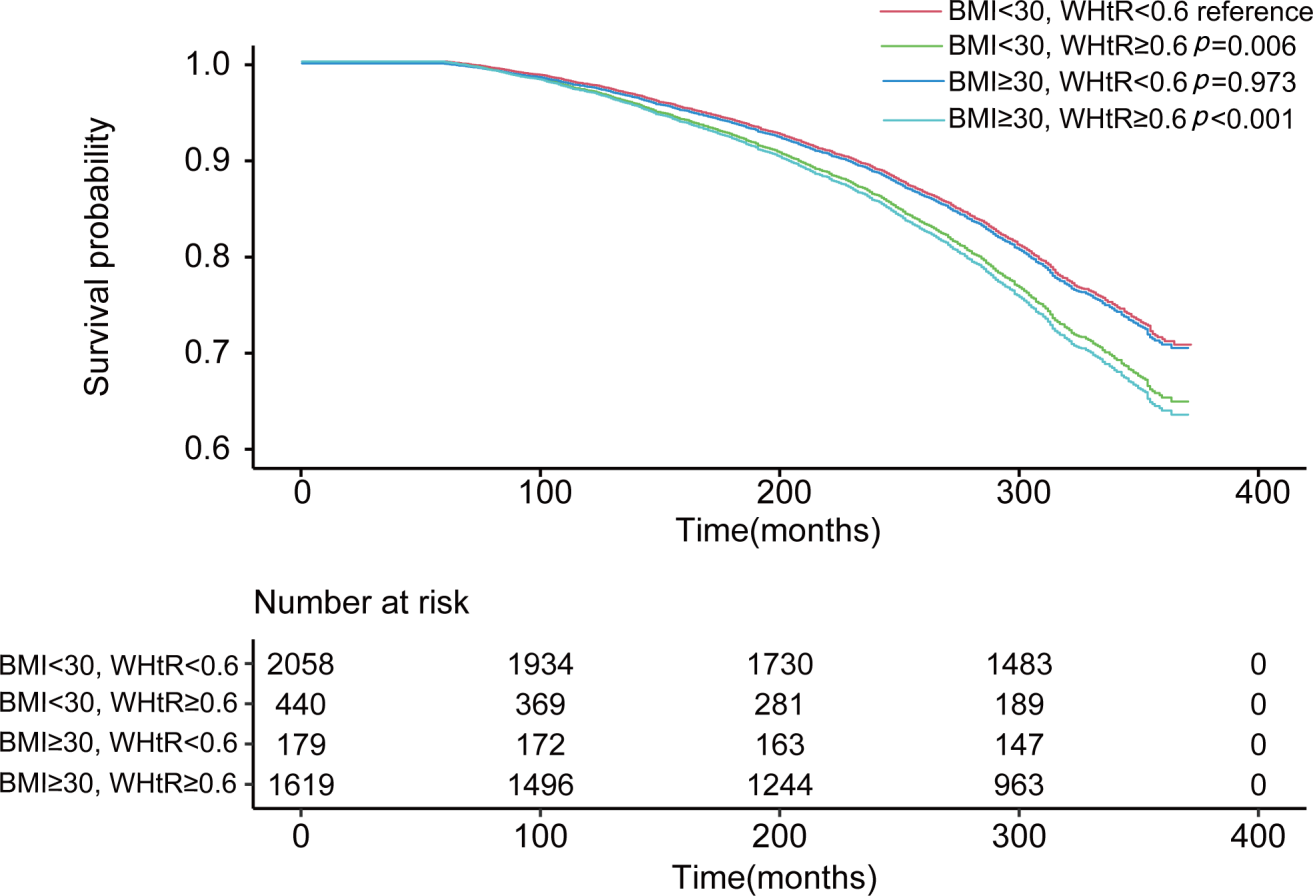
The combined effect of body mass index and the waist-to-height ratio on mortality. Adjusted for age, sex, ethnicity, and smoking status and excluding the first 5 years of follow-up.

**Table 4** shows the multivariate Cox proportional hazards analysis results, which were used to evaluate the impact of combined BMI+WHtR categories on all-cause mortality risk, with the “Non-obesity group” used as a reference. The analysis across the three models revealed increased mortality risks in the “Simple abdominal obesity” (BMI <30 kg/m², WHtR ≥0.6) and “Double obesity” (BMI ≥30 kg/m², WHtR ≥0.6) groups, while the “Simple general obesity” (BMI ≥30 kg/m², WHtR <0.6) group did not show a heightened risk (p > 0.05). And the survival curves got the same result (**Figure 5**). However, when we further compared the risk of death between the “Simple general obesity” and “Simple abdominal obesity” groups, there was no significant difference, with a p value of 0.221 (**Supplementary Table S4**).

**Table 4:** Mortality risk for the four groups based on body mass index and waist-to-height ratio.

	BMI < 30, WHtR < 0.6	BMI < 30, WHtR ≥ 0.6	BMI ≥ 30, WHtR < 0.6	BMI ≥ 30, WHtR ≥ 0.6
All-cause mortality				
Deaths/Participants	663/2058 (32.22%)	281/440 (63.86%)	40/179 (22.35%)	764/1619 (47.19%)
Event rate per 1000 person-years	13.45	33.04	8.84	21.06
HR (95% CI)^a^	Reference	1.204 (1.041, 1.393)*	0.862 (0.625, 1.187)	1.202 (1.081, 1.336)***
HR (95% CI)^b^	Reference	1.250 (1.065, 1.466)**	1.006 (0.713, 1.420)	1.311 (1.169, 1.470)***
HR (95% CI)^c^	Reference	1.221 (1.040, 1.434) *	1.022 (0.724, 1.443)	1.286 (1.146, 1.443) ***

BMI, body mass index; WHtR, waist-to-height ratio; CI, confidence interval; HR, hazard ratio.

a Adjusted for age, sex and ethnicity.

b Adjusted for age, sex, ethnicity, smoking status and excluding the first 5 years of follow-up.

c Adjusted for age, sex, ethnicity, smoking status, known chronic disease at baseline (hypertension, diabetes, heart failure, heart attack, stroke, asthma, chronic bronchitis, skin cancer, other cancers) and excluding the first 5 years of follow-up.

*P* value *P<0.05 **P<0.01 ***P<0.001.

### 3.6 Subgroup analysis

#### 3.6.1 Body mass index

Subgroup analyses by sex, age, and ethnicity were conducted to explore potential effect modifications on the relationships among BMI, WHtR, and mortality. The findings indicated that overweight (HR 0.718, 95% CI 0.717–0.720) and obese class I (HR 0.810, 95% CI 0.808–0.812) males had a reduced mortality risk compared to those of normal weight individuals. In contrast, weight gain in females is often correlated with increased mortality risk (**Supplementary Table S5**). Participants aged ≥65 years with class I obesity (HR 0.919, 95% CI 0.917–0.922) also had a lower mortality risk than those with a normal weight (**Supplementary Table S6**). Among non-Hispanic black individuals, overweight (HR 0.924, 95% CI 0.919–0.928) and obese class I (HR 0.960, 95% CI 0.955–0.965) individuals had a lower mortality risk than normal weight individuals, and among Mexican Americans, those who were overweight (HR 0.875, 95% CI 0.869–0.882) had the lowest mortality risk (**Supplementary Table S7**).

#### 3.6.2 Waist-to-height ratio

Regarding BMI, men in quartile 2 (HR 0.836, 95% CI 0.834–0.838) presented the lowest mortality risk, whereas in women, mortality risk increased with increased WHtR (**Supplementary Table S8**). Among participants aged ≥65 years, those in quartile 2 (HR 0.881, 95% CI 0.878–0.884) faced a lower mortality risk than those in quartile 1 (**Supplementary Table S9**). Across all the ethnic groups, Quartile 1 presented the lowest mortality risk (**Supplementary Table S10**).

## 4 Discussion

In our cohort study, we scrutinized the associations between BMI and WHtR with mortality risk in patients with NAFLD, considering potential confounders and reverse causation. Post-adjustment for smoking and reverse causation, the supposed BMI-related “obesity−survival paradox” among NAFLD patients dissipated. It became apparent that increased adiposity correlated with increased mortality risk, especially when the WHtR was considered, which demonstrated a pronounced dose−response relationship with mortality risk across its entire spectrum. Conversely, BMI exhibited a U-shaped correlation with mortality risk, with the nadir risk situated within the normal weight bracket (18.5−25 kg/m^2^).

In analyses with minimal adjustments (considering only age, sex, and ethnicity), being overweight or obese, as defined by standard BMI categories, was linked to a reduced mortality risk compared with normal weight, in accordance with previous reports (5, 10, 11). However, many earlier studies did not account for smoking and reverse causation (19, 20). Upon making these adjustments, the “survival paradox” associated with elevated BMI vanished. The confounding effects of smoking and reverse causality are significant biases when the relationship between weight status and mortality is estimated. Our interpretation of these findings suggests that the obesity paradox is more an artifact of reverse causation and smoking-related confounding than a genuine biological occurrence, contrary to what some prior research has suggested (21). Our conclusions also align with more rigorous analyses from a 2016 meta-analysis encompassing 239 prospective studies and a 2016 review that sought to curtail the reverse causality impact (14, 22). The pervasive nature of the “obesity−survival paradox,” despite its rarity in epidemiological phenomena, prompts further inquiry. We hypothesize that the inverse relationship between obesity and smoking—another key mortality risk factor—partially elucidates this enigma (23). When two risk factors exhibit a positive correlation, neglecting or inaccurately measuring one can lead to an inflated estimation of the other’s effect, resulting in an amplification rather than a paradox of the anticipated association (24). Overstatements in research findings may not face as much scrutiny as the “obesity–survival anomaly” because they align with preconceived notions; this is typical for overstatements to surface in observational research since numerous risk determinants often show positive correlations. Nonetheless, a paradox arises when the correlation observed contradicts what was anticipated. Such instances are more probable when risk determinants have negative correlations and if one is overlooked or inaccurately assessed. We advocate that subsequent research on obesity anomalies should concentrate more on the inverse relationship between tobacco use and obesity due to its potential to skew results. Additionally, we disregarded the initial five-year period from our study to consider pre-existing conditions that could cause death within the following five years, thus avoiding the possibility of reverse causality. Under these circumstances, a reduced BMI at the beginning may be a result, not a cause, of existing health issues. Interestingly, after further adjustment for various chronic conditions (hypertension, diabetes, heart failure, heart attack, stroke, asthma, chronic bronchitis, skin cancer, other cancers), we noticed no notable change in the association between body measurements and mortality risk. This lack of change could be due to the relatively small number of chronic conditions within our study group. Furthermore, the significant decrease in reverse causality, achieved by omitting participants with less than five years of follow-up, may explain this outcome.

Importantly, when we combined BMI and the waist-to-height ratio to assess their impact on mortality risk, we detected a significantly increased risk of death in individuals with greater adiposity, as indicated by their waist-to-height ratio. Conversely, this link was not as apparent when BMI alone was considered, suggesting that general obesity, as measured by BMI alone, may not be as predictive of mortality risk when the WHtR is within the normal range, indicating the potential limitations of BMI as a sole indicator of health risk. These findings highlight the complex relationship between adiposity, as measured by BMI and WHtR, and mortality risk. We suggest that central obesity, as indicated by a high WHtR, may be a more potent predictor of all-cause mortality than general obesity measured by BMI alone; this underscores the importance of considering both BMI and WHtR in assessing mortality risk and suggests that interventions targeting abdominal obesity may be particularly beneficial in reducing the risk of all-cause mortality.

In our investigation, we discovered that, even after accounting for smoking habits and reverse causality, men who were overweight or obese had a reduced mortality risk compared with those with a normal weight. This finding aligns with a 2021 study examining gender differences in the “obesity paradox” (25). Similarly, men in the second quartile for WHtR presented a lower mortality risk than those in other quartiles did. In older adults aged 65 years and above, being overweight and having a WHtR in the second quartile correlated with the lowest mortality risk, echoing the results of a 2022 study (26). Across various ethnicities, we observed that black Mexicans and Mexican Americans with overweight or obesity had the lowest mortality risk when evaluated via BMI, whereas those in the first quartile for WHtR had the lowest risk when assessed via WHtR. The gathered data strongly indicate a link between increased adiposity in individuals with NAFLD and a greater risk of death from all causes. Research has also shown that obese individuals are more susceptible to a range of comorbidities, such as diabetes, high blood pressure, atrial fibrillation, and sleep apnea, than their non-obese counterparts (27, 28). Therefore, there is a strong argument for advocating weight reduction programs for obese individuals.

This study has several strengths. First, recently, an increasing number of studies have focused on lean NAFLD (10), as highlighted by Albert Do et al. (11), who demonstrated that lean individuals with NAFLD are associated with a greater risk of adverse liver events and mortality. Addressing this phenomenon, our research reveals that part of the explanation can be attributed to smoking and reverse causation, making a significant contribution to the existing body of literature by being one of the first to investigate the “obesity paradox” within the context of NAFLD. Moreover, compared with previous studies on the obesity paradox (12, 16), we have extended our investigation to include the WHtR, a relatively novel indicator, and delved into the complex relationships among BMI, the WHtR, and mortality, offering new insights into how these metrics influence outcomes in NAFLD patients. However, this study has several limitations. Measurements of abdominal dimensions, particularly waist circumference, are more susceptible to inaccuracies than BMI is, especially when the former is measured by different individuals (29). Additionally, the study’s analysis does not account for any changes in weight or waist circumference during the follow-up period. It’s also important to note that, our results are not applicable to individuals with a low BMI or waist-to-height ratio, as only 71 participants were underweight (BMI < 18.5 kg/m^2^), and 39 had a waist-to-height ratio < 0.40.

## 5 Conclusion

In our substantial group of patients diagnosed with NAFLD, the apparent “obesity−survival paradox” associated with BMI was clarified after careful adjustment for various predictive factors. Moreover, a closer look at the combined effects of BMI and the waist-to-height ratio on mortality revealed that increased adiposity, as suggested by the waist-to-height ratio, markedly increased the risk of death. This correlation was less evident when BMI alone was considered. Both BMI and the waist-to-height ratio, which are indicators of obesity, consistently indicate that obesity tends to increase mortality risk.

## Data Availability

Publicly available datasets were analyzed in this study. This data can be found here: https://www.cdc.gov/nchs/nhanes/index.htm.
