# Supplementary material for: Body mass index and waist-to-height ratio effect on mortality in non-alcoholic fatty liver: revisiting the obesity paradox
Source: Front Endocrinol (Lausanne). 2024 Dec 6;15:1419715. doi: 10.3389/fendo.2024.1419715 (PMC11658989; doi:10.3389/fendo.2024.1419715)
Supplement: Supplementary file 1 [file Table1.docx]

**Supplementary Table S1 Steatogenic medication for more than 3 months**

| **Steatogenic Medications** | | **Number** |
| --- | --- | --- |
| **Glucocorticoids** | **Prednisone, Prednisolone** | **22** |
|  | **Dexamethasone** | **2** |
|  | **Hydrocortisone** | **1** |
|  | **Methylprednisolone** | **2** |
| **Tamoxifen** | | **3** |
| **Amiodarone** | | **4** |
| **Methotrexate** | | **2** |
| **Divalproex Sodium** | | **6** |

**Supplementary Table S2 Baseline characteristics according to body mass index**

| **Clinical characteristics** | **Underweight**  **BMI < 18.5** | **Normal weight BMI 18.5 - 25** | **Overweight**  **BMI 25 - 30** | **Obesity class I BMI 30 - 35** | **Obesity class II /III**  **BMI ≥ 35** | ***P* value** |
| --- | --- | --- | --- | --- | --- | --- |
| Sample N | 71 | 1021 | 1486 | 1066 | 800 |  |
| Weighted N | 949850.98 | 11852133.16 | 15064787.28 | 10633887.39 | 7751343.42 |  |
| Age (years) | 37.12 ± 13.08 | 39.24 ± 14.93 | 48.15 ± 14.48 | 48.65 ± 13.96 | 45.15 ± 14.09 | < 0.0001 |
| Sex, (%) |  |  |  |  |  | < 0.0001 |
| Male | 22.66 | 40.49 | 62.34 | 56.43 | 32.82 |  |
| Female | 77.34 | 59.51 | 37.66 | 43.57 | 67.18 |  |
| Ethnicity, (%) |  |  |  |  |  | 0.0029 |
| Non-Hispanic White | 74.29 | 75.07 | 76.13 | 74.89 | 73.04 |  |
| Non-Hispanic Black | 10.96 | 9.74 | 7.93 | 8.54 | 13.47 |  |
| Mexican American | 2.61 | 5.76 | 7.78 | 7.57 | 6.35 |  |
| Other | 12.14 | 9.44 | 8.16 | 9.01 | 7.14 |  |
| Poverty income ratio category, (%) |  |  |  |  |  | < 0.0001 |
| ≤1.3 | 13.84 | 15.77 | 15.73 | 17.81 | 21.82 |  |
| >1.3, ≤3.5 | 48.34 | 43.86 | 43.07 | 43.91 | 49.45 |  |
| >3.5 | 31.46 | 36.34 | 35.79 | 32.52 | 23.76 |  |
| Not recorder | 6.36 | 4.03 | 5.41 | 5.76 | 4.97 |  |
| Education level, (%) |  |  |  |  |  | < 0.0001 |
| ≤high school | 59.69 | 51.32 | 64.79 | 64.63 | 71.65 |  |
| >high school degree | 40.31 | 48.51 | 34.88 | 35.27 | 28.06 |  |
| Not recorder | 0 | 0.18 | 0.33 | 0.11 | 0.29 |  |
| Smoke state, (%) |  |  |  |  |  | < 0.0001 |
| Never | 45.66 | 47.80 | 45.18 | 43.13 | 50.40 |  |
| Previous | 14.94 | 21.89 | 32.51 | 35.58 | 31.42 |  |
| Current | 39.39 | 30.31 | 22.30 | 21.29 | 18.19 |  |
| Height (cm) | 165.33 ± 7.87 | 167.14 ± 9.31 | 169.79 ± 10.00 | 169.17 ± 10.17 | 166.81 ± 10.34 | < 0.0001 |
| Weight (cm) | 48.42 ± 5.02 | 62.27 ± 9.25 | 79.32 ± 10.54 | 91.99 ± 11.56 | 112.75 ± 20.09 | < 0.0001 |
| Body mass index (kg/m^2^ ) | 17.67 ± 0.67 | 22.19 ± 1.80 | 27.40 ± 1.42 | 32.03 ± 1.35 | 40.38 ± 5.25 | < 0.0001 |
| Waist Circumference (cm) | 69.72 ± 4.37 | 80.00 ± 8.74 | 97.05 ± 7.47 | 107.05 ± 7.50 | 120.67 ± 12.44 | < 0.0001 |
| Waist-to-height ratio | 0.42 ± 0.03 | 0.48 ± 0.05 | 0.57 ± 0.04 | 0.63 ± 0.04 | 0.72 ± 0.07 | < 0.0001 |
| Platelet (10^9^/L) | 257.77 ± 81.23 | 270.07 ± 71.81 | 269.10 ± 67.53 | 275.52 ± 70.54 | 285.20 ± 73.63 | < 0.0001 |
| Triglycerides (mmol/L) | 1.16 ± 1.20 | 1.33 ± 1.02 | 2.31 ± 1.66 | 2.38 ± 1.73 | 2.40 ± 2.15 | < 0.0001 |
| High-density lipoprotein cholesterol (mmol/L) | 1.56 ± 0.53 | 1.38 ± 0.42 | 1.15 ± 0.35 | 1.11 ± 0.35 | 1.10 ± 0.30 | < 0.0001 |
| Glucose (mmol/L) | 4.75 ± 0.62 | 5.17 ± 1.50 | 5.78 ± 2.17 | 5.99 ± 2.24 | 6.43 ± 3.02 | < 0.0001 |
| Aspartate aminotransferase (mmol/L) | 22.87 ± 13.82 | 20.64 ± 12.83 | 23.31 ± 14.70 | 23.32 ± 10.94 | 24.58 ± 15.32 | < 0.0001 |
| Alanine aminotransferase (mmol/L) | 16.03 ± 11.73 | 16.24 ± 13.29 | 22.20 ± 16.71 | 23.76 ± 15.71 | 25.47 ± 21.00 | < 0.0001 |
| Gamma-glutamyl transferase(mmol/L) | 17.76 ± 39.61 | 24.17 ± 36.18 | 35.98 ± 37.88 | 40.79 ± 45.93 | 38.92 ± 42.64 | < 0.0001 |
| Alkaline phosphatase (U/L) | 73.61 ± 22.10 | 77.81 ± 28.72 | 86.42 ± 26.50 | 90.94 ± 31.44 | 89.58 ± 28.55 | < 0.0001 |
| Albumin (g/L) | 43.06 ± 3.72 | 42.33 ± 3.38 | 42.07 ± 3.26 | 41.50 ± 3.12 | 40.18 ± 3.47 | < 0.0001 |
| Globulin (g/L) | 32.23 ± 4.80 | 31.01 ± 4.17 | 31.68 ± 4.11 | 32.20 ± 3.93 | 33.54 ± 4.26 | < 0.0001 |
| Glycosylated hemoglobin (%) | 5.08 ± 0.62 | 5.22 ± 0.86 | 5.55 ± 1.12 | 5.72 ± 1.22 | 6.03 ± 1.53 | < 0.0001 |
| Heart failure disease, n (%) | 0 | 1.20 | 3.23 | 3.20 | 2.88 | 0.0041 |
| Heart attack disease, n (%) | 0.26 | 2.12 | 5.09 | 5.68 | 3.45 | < 0.0001 |
| Stroke disease, (%) | 1.88 | 0.70 | 2.22 | 2.71 | 2.15 | 0.0099 |
| Asthma disease, (%) | 9.91 | 3.42 | 2.95 | 6.69 | 10.16 | < 0.0001 |
| Chronic bronchitis disease, (%) | 6.74 | 3.46 | 4.44 | 4.41 | 6.37 | 0.0481 |
| Skin cancer disease, (%) | 4.20 | 2.84 | 3.47 | 4.42 | 3.40 | 0.3883 |
| Other cancer disease, (%) | 16.77 | 1.88 | 2.81 | 4.53 | 3.88 | < 0.0001 |
| Hypertension, (%) | 3.87 | 11.37 | 28.77 | 37.46 | 50.56 | < 0.0001 |
| Diabetes, (%) | 0.94 | 3.32 | 10.31 | 15.63 | 22.77 | < 0.0001 |
| Hepatic steatosis ultrasound examination, (%) |  |  |  |  |  | < 0.0001 |
| Mild | 56.29 | 61.97 | 37.32 | 33.43 | 22.32 |  |
| Moderate | 41.71 | 28.55 | 41.53 | 44.37 | 50.14 |  |
| Severe | 2.00 | 9.49 | 21.16 | 22.20 | 27.54 |  |
| FIB-4 category, (%) |  |  |  |  |  | < 0.0001 |
| <1.3 | 76.64 | 79.60 | 74.50 | 76.79 | 81.16 |  |
| ≥1.3, <2.67 | 16.53 | 12.87 | 20.58 | 17.17 | 12.07 |  |
| ≥2.67 | 0.9 | 1.12 | 0.93 | 1.45 | 1.73 |  |
| Not recorder | 5.93 | 6.41 | 3.98 | 4.59 | 5.04 |  |
| All-cause mortality, (%) | 28.34 | 23.93 | 37.99 | 41.62 | 44.73 | < 0.0001 |

**Supplementary Table S3 Baseline characteristics according to quartile of waist-to-height ratio**

| **Clinical characteristics** | **Quartile 1**  **0.35~0.53** | **Quartile 2**  **0.53~0.60** | **Quartile 3**  **0.60~0.66** | **Quartile 4**  **0.66~1.00** | ***P* value** |
| --- | --- | --- | --- | --- | --- |
| Sample N | 1074 | 1074 | 1074 | 1074 |  |
| Weighted N | 13671247.36 | 11405143.93 | 10497215.05 | 9364901.97 |  |
| Age (years) | 36.86 ± 12.90 | 46.80 ± 14.11 | 50.39 ± 14.25 | 49.43 ± 14.32 | <0.0001 |
| Sex, (%) |  |  |  |  | <0.0001 |
| Male | 43.28 | 64.59 | 56.06 | 32.06 |  |
| Female | 56.72 | 35.41 | 43.94 | 67.94 |  |
| Ethnicity, (%) |  |  |  |  | 0.0002 |
| Non-Hispanic White | 74.54 | 76.71 | 74.38 | 74.70 |  |
| Non-Hispanic Black | 10.89 | 7.55 | 7.59 | 11.06 |  |
| Mexican American | 5.12 | 7.76 | 7.48 | 7.64 |  |
| Other | 9.44 | 7.97 | 10.54 | 6.60 |  |
| Poverty income ratio category, (%) |  |  |  |  | <0.0001 |
| ≤1.3 | 15.23 | 14.04 | 17.44 | 23.48 |  |
| >1.3, ≤3.5 | 43.05 | 45.84 | 41.78 | 49.36 |  |
| >3.5 | 37.41 | 35.91 | 33.90 | 21.19 |  |
| Not recorder | 4.31 | 4.21 | 6.88 | 5.96 |  |
| Education level, (%) |  |  |  |  | <0.0001 |
| ≤high school | 50.99 | 64.07 | 65.18 | 74.62 |  |
| >high school degree | 48.64 | 35.82 | 34.68 | 25.15 |  |
| Not recorder | 0.37 | 0.11 | 0.14 | 0.24 |  |
| Smoke state, (%) |  |  |  |  | <0.0001 |
| Never | 48.97 | 45.86 | 42.28 | 44.70 |  |
| Previous | 19.81 | 33.53 | 35.27 | 35.11 |  |
| Current | 31.21 | 20.61 | 22.45 | 20.19 |  |
| Height (cm) | 168.31 ± 9.67 | 170.54 ± 9.67 | 168.81 ± 9.79 | 165.15 ± 10.02 | <0.0001 |
| Weight (cm) | 64.12 ± 12.22 | 80.67 ± 12.70 | 89.21 ± 14.80 | 103.97 ± 21.92 | <0.0001 |
| Body mass index (kg/m^2^ ) | 22.48 ± 2.69 | 27.58 ± 2.46 | 31.11 ± 3.02 | 37.89 ± 5.97 | <0.0001 |
| Waist Circumference (cm) | 79.62 ± 8.82 | 96.53 ± 6.08 | 105.47 ± 6.57 | 119.25 ± 11.49 | <0.0001 |
| Waist-to-height ratio | 0.47 ± 0.04 | 0.57 ± 0.02 | 0.62 ± 0.02 | 0.72 ± 0.06 | <0.0001 |
| Platelet (10^9^/L) | 266.36 ± 68.15 | 270.19 ± 71.46 | 273.85 ± 69.62 | 287.56 ± 73.75 | <0.0001 |
| Triglycerides (mmol/L) | 1.33 ± 1.04 | 2.39 ± 1.85 | 2.40 ± 1.68 | 2.37 ± 1.99 | <0.0001 |
| High-density lipoprotein cholesterol (mmol/L) | 1.36 ± 0.42 | 1.14 ± 0.36 | 1.12 ± 0.33 | 1.13 ± 0.35 | <0.0001 |
| Glucose (mmol/L) | 5.06 ± 1.17 | 5.66 ± 2.08 | 6.00 ± 2.17 | 6.55 ± 3.04 | <0.0001 |
| Aspartate aminotransferase (mmol/L) | 20.83 ± 12.73 | 23.18 ± 13.85 | 23.71 ± 14.07 | 24.29 ± 14.23 | <0.0001 |
| Alanine aminotransferase (mmol/L) | 17.15 ± 13.80 | 22.05 ± 15.84 | 24.43 ± 19.89 | 23.60 ± 17.29 | <0.0001 |
| Gamma-glutamyl transferase(mmol/L) | 25.43 ± 39.74 | 34.44 ± 38.73 | 39.31 ± 39.30 | 40.76 ± 45.76 | <0.0001 |
| Alkaline phosphatase (U/L) | 76.35 ± 25.39 | 85.36 ± 27.22 | 89.57 ± 26.74 | 92.97 ± 34.31 | <0.0001 |
| Albumin (g/L) | 42.57 ± 3.33 | 42.04 ± 3.31 | 41.63 ± 3.12 | 40.23 ± 3.30 | <0.0001 |
| Globulin (g/L) | 31.06 ± 4.14 | 31.56 ± 4.18 | 32.40 ± 4.01 | 33.30 ± 4.22 | <0.0001 |
| Glycosylated hemoglobin, (%) | 5.15 ± 0.68 | 5.51 ± 1.15 | 5.68 ± 1.14 | 6.11 ± 1.54 | <0.0001 |
| Heart failure disease, (%) | 0.81 | 1.65 | 4.58 | 3.46 | <0.0001 |
| Heart attack disease, (%) | 1.42 | 3.26 | 7.62 | 4.75 | <0.0001 |
| Stroke disease, (%) | 0.52 | 1.43 | 2.71 | 3.18 | <0.0001 |
| Asthma disease, (%) | 3.35 | 2.02 | 6.50 | 10.21 | <0.0001 |
| Chronic bronchitis disease, (%) | 3.06 | 2.04 | 7.14 | 6.96 | <0.0001 |
| Skin cancer disease, (%) | 1.88 | 4.22 | 4.62 | 3.97 | 0.0012 |
| Other cancer disease, (%) | 2.95 | 2.54 | 3.57 | 4.92 | 0.0219 |
| Hypertension, (%) | 10.09 | 25.53 | 39.35 | 50.63 | <0.0001 |
| Diabetes, (%) | 1.73 | 8.91 | 16.19 | 23.69 | <0.0001 |
| Hepatic steatosis ultrasound examination, (%) |  |  |  |  | <0.0001 |
| Mild | 61.23 | 41.91 | 28.04 | 22.96 |  |
| Moderate | 28.80 | 40.64 | 46.17 | 50.01 |  |
| Severe | 9.96 | 17.45 | 25.79 | 27.04 |  |
| FIB-4 category, (%) |  |  |  |  | <0.0001 |
| <1.3 | 82.71 | 76.30 | 75.75 | 74.26 |  |
| ≥1.3, <2.67 | 10.42 | 18.56 | 19.48 | 18.64 |  |
| ≥2.67 | 0.75 | 0.80 | 1.38 | 2.39 |  |
| Not recorder | 6.11 | 4.34 | 3.39 | 4.71 |  |
| All-cause mortality, (%) | 17.65 | 33.73 | 46.81 | 53.46 | <0.0001 |

**Supplementary Table S4 Post hoc tests for multiple Kaplan-Meier survival curves**

| **All-cause mortality** | **versus BMI < 30, WHtR < 0.6**  ***P*-value** | **versus BMI < 30, WHtR ≥ 0.6**  ***P*-value** | **Versus BMI ≥ 30, WHtR < 0.6**  ***P*-value** |
| --- | --- | --- | --- |
| **BMI < 30, WHtR ≥ 0.6** | 0.006 | - | - |
| **BMI ≥ 30, WHtR < 0.6** | 0.973 | 0.221 | - |
| **BMI ≥ 30, WHtR ≥ 0.6** | <0.001 | 0.603 | 0.129 |

**Supplementary Table S5 Mortality risk for the non-alcoholic fatty liver disease according to body mass index by sex**

|  | **Deaths/Participants, n %** | **Male HR (95% CI)** | **Deaths/Participants, n %** | **Female HR (95% CI)** |
| --- | --- | --- | --- | --- |
| **All-cause mortality** |  |  |  |  |
| BMI < 18.5 | 3/14（7.2%） | 0.426 (0.418, 0.433) | 10/45 (26.2%) | 3.511 (3.493, 3.529) |
| BMI 18.5 - 25 | 150/398（30.1%） | Reference | 115/534 (16.3%) | Reference |
| BMI 25 - 30 | 327/752（34.5%） | 0.718 (0.717, 0.720) | 227/605 (39.4%) | 1.554 (1.550, 1.557) |
| BMI 30 - 35 | 205/479（39.5%） | 0.810 (0.808, 0.812) | 202/487 (42.8%) | 1.503 (1.499, 1.507) |
| BMI ≥ 35 | 102/211（44.9%） | 1.562 (1.558, 1.566) | 221/524 (42.6%) | 2.430 (2.424, 2.436) |

BMI, body mass index; CI, confidence interval; HR, hazard ratio.

Adjusted for age, sex, ethnicity, smoking status and excluding the first 5 years of follow-up.

**Supplementary Table S6 Mortality risk for the non-alcoholic fatty liver disease according to body mass index by age**

|  | **Deaths/Participants, n %** | **Age < 65 HR (95% CI)** | **Deaths/Participants, n %** | **Age ≥ 65 HR (95% CI)** |
| --- | --- | --- | --- | --- |
| **All-cause mortality** |  |  |  |  |
| BMI < 18.5 | 10/56（18.1%） | 1.260 (1.253, 1.267) | 3/3 (100%) | 4.432 (4.388, 4.477) |
| BMI 18.5 - 25 | 161/817（14.6%） | Reference | 104/115 (94.0%) | Reference |
| BMI 25 - 30 | 312/1094（25.4%） | 1.845 (1.841, 1.849) | 242/263 (95.3%) | 1.120 (1.117, 1.122) |
| BMI 30 - 35 | 246/789（31.1%） | 2.342 (2.338, 2.347) | 161/177 (92.2%) | 0.919 (0.917, 0.922) |
| BMI ≥ 35 | 239/643（37.8%） | 3.054 (3.047, 3.060) | 84/92 (95.2%) | 1.358 (1.354, 1.363) |

BMI, body mass index; CI, confidence interval; HR, hazard ratio.

Adjusted for age, sex, ethnicity, smoking status and excluding the first 5 years of follow-up.

**Supplementary Table S7 Mortality risk for the non-alcoholic fatty liver disease according to body mass index by ethnicity**

|  | **Deaths/Participants, n %** | **Non-Hispanic White** | **Deaths/Participants, n %** | **Non-Hispanic Black** | **Deaths/Participants, n %** | **Mexican American** | **Deaths/Participants, n %** | **Other** |
| --- | --- | --- | --- | --- | --- | --- | --- | --- |
| **All-cause mortality** |  |  |  |  |  |  |  |  |
| BMI < 18.5 | 8/24（28.0%） | 2.474 (2.462, 2.487) | 3/20（11.5%） | 0.817 (0.801, 0.834) | 2/12（19.3%） | 1.706 (1.655, 1.760) | 0/3（0%） | - |
| BMI 18.5 - 25 | 114/340（23.3%） | Reference | 73/252（26.2%） | Reference | 73/297（17.3%） | Reference | 5/43（8.0%） | Reference |
| BMI 25 - 30 | 247/478（38.5%） | 1.002 (1.000, 1.003) | 105/261（35.1%） | 0.924 (0.919, 0.928) | 186/567（22.2%） | 0.875 (0.869, 0.882) | 187/51（31.6%） | 3.116 (3.093, 3.140) |
| BMI 30 - 35 | 180/332（44.5%） | 1.082 (1.080, 1.084) | 89/207（39.7%） | 0.960 (0.955, 0.965) | 127/387（25.0%） | 1.028 (1.020, 1.036) | 11/40（25.2%） | 2.327 (2.309, 2.346) |
| BMI ≥ 35 | 130/238（47.3%） | 2.151 (2.147, 2.155) | 103/237（49.0%） | 1.148 (1.142, 1.154) | 80/236（26.9%） | 1.280 (1.269, 1.290) | 10/24（28.4%） | 3.381 (3.352, 3.410) |

BMI, body mass index; CI, confidence interval; HR, hazard ratio.

Adjusted for age, sex, ethnicity, smoking status and excluding the first 5 years of follow-up.

**Supplementary Table S8 Mortality risk for the non-alcoholic fatty liver disease according to waist-to-height ratio by sex**

|  | **Deaths/Participants, n %** | **Male HR (95% CI)** | **Deaths/Participants, n %** | **Female HR (95% CI)** |
| --- | --- | --- | --- | --- |
| **All-cause mortality** |  |  |  |  |
| Quartile 1 | 117/440（19.8%） | Reference | 76/543 (13.1%) | Reference |
| Quartile 2 | 235/582（33.0%） | 0.836 (0.834, 0.838) | 121/400 (31.5%) | 1.367 (1.363, 1.371) |
| Quartile 3 | 251/501（46.0%） | 1.150 (1.147, 1.153) | 206/481 (43.6%) | 1.702 (1.698, 1.706) |
| Quartile 4 | 156/281（56.0%） | 1.568 (1.564, 1.572) | 339/702 (50.8%) | 2.356 (2.351, 2.362) |

CI, confidence interval; HR, hazard ratio; WHtR, waist-to-height ratio.

Adjusted for age, sex, ethnicity, smoking status and excluding the first 5 years of follow-up.

**Supplementary Table S9 Mortality risk for the non-alcoholic fatty liver disease according to waist-to-height ratio by age**

|  | **Deaths/Participants, n %** | **Age < 65 HR (95% CI)** | **Deaths/Participants, n %** | **Age ≥ 65 HR (95% CI)** |
| --- | --- | --- | --- | --- |
| **All-cause mortality** |  |  |  |  |
| Quartile 1 | 140/924（11.9%） | Reference | 53/59 (91.4%) | Reference |
| Quartile 2 | 206/822（22.8%） | 2.020 (2.015, 2.024) | 150/160 (92.3%) | 0.881 (0.878, 0.884) |
| Quartile 3 | 262/771（32.7%） | 3.058 (3.051, 3.064) | 195/211 (97.1%) | 1.186 (1.183, 1.190) |
| Quartile 4 | 323/789（44.4%） | 4.798 (4.789, 4.808) | 172/194 (93.9%) | 1.283 (1.279, 1.287) |

CI, confidence interval; HR, hazard ratio; WHtR, waist-to-height ratio.

Adjusted for age, sex, ethnicity, smoking status and excluding the first 5 years of follow-up.

**Supplementary Table S10 Mortality risk for the non-alcoholic fatty liver disease according to waist-to-height ratio by ethnicity**

|  | **Deaths/Participants, n %** | **Non-Hispanic White** | **Deaths/Participants, n %** | **Non-Hispanic Black** | **Deaths/Participants, n %** | **Mexican American** | **Deaths/Participants, n %** | **Other** |
| --- | --- | --- | --- | --- | --- | --- | --- | --- |
| **All-cause mortality** |  |  |  |  |  |  |  |  |
| **Quartile 1** | 85/351（16.9%） | Reference | 69/315（21.3%） | Reference | 36/274（10.8%） | Reference | 3/43（5.0%） | Reference |
| **Quartile 2** | 164/357（34.1%） | 1.017 (1.015, 1.019) | 83/194（39.3%） | 1.065 (1.060, 1.071) | 102/398（17.8%） | 1.125 (1.115, 1.136) | 7/33（25.1%） | 2.218 (2.197, 2.239) |
| **Quartile 3** | 209/347（50.0%） | 1.440 (1.437, 1.443) | 77/178（36.5%） | 1.005 (1.000, 1.011) | 156/413（27.0%） | 1.270 (1.259, 1.281) | 15/44（28.9%） | 2.731 (2.707, 2.756) |
| **Quartile 4** | 204/324（56.9%） | 2.067 (2.064, 2.071) | 118/244（44.8%） | 1.211 (1.204, 1.217) | 156/379（32.9%） | 1.673 (1.658, 1.687) | 17/36（40.5%） | 3.108 (3.079, 3.138) |

CI, confidence interval; HR, hazard ratio; WHtR, waist-to-height ratio.

Adjusted for age, sex, ethnicity, smoking status and excluding the first 5 years of follow-up.
